# First record of *Phlebotomus* (*Larroussius*) *orientalis* (Parrot, 1936) (Diptera: Psychodidae) in Israel: phylogeographic placement and implications for leishmaniasis surveillance

**DOI:** 10.1186/s13071-026-07358-5

**Published:** 2026-03-29

**Authors:** Debora Diaz, Edwin Kniha, Stephan Koblmüller, Liora Studentsky, Shirly Lea Elbaz, Ira Ben Avi, Simcha Shilo, Shira Kalmus, Fouad Akad, Itay Naveh, Shay Reicher, Maya Davidovich-Cohen, Laor Orshan, Oscar David Kirstein

**Affiliations:** 1 Public Health Laboratories, Jerusalem (PHL-J), Public Health Services (PHS), Ministry of Health (MOH), Jerusalem, Israel; 2 Institute of Specific Prophylaxis and Tropical Medicine, Center for Pathophysiology, Infectiology and Immunology, Medical University of Vienna, Vienna, Austria; 3 Institute of Biology, University of Graz, Graz, Austria; 4 Nature and Parks Authority, Jerusalem, Israel; 5 Division of Pest Control and Pesticides, Ministry of Environmental Protection, Jerusalem, Israel; 6 Public Health Laboratories, Tel Aviv, Public Health Services, Ministry of Health (MOH), Tel Aviv, Israel

**Keywords:** *Phlebotomusorientalis*, Sand flies, Barcoding, Haplotyping, Divergence time estimates, Negev Desert, Leishmaniasis

## Abstract

**Background:**

Phlebotomine sand flies (Diptera: Psychodidae) are the principal vectors of *Leishmania* spp., the causative agents of leishmaniasis. Since 2008, the Ministry of Health and the Ministry of Environmental Protection of Israel have conducted nationwide, periodic sand fly surveys. Initially, these surveys focused on localities with known endemic transmission of cutaneous leishmaniasis, but in recent years, entomological trapping has expanded to areas where prior information on sand flies was unavailable. Here we report the first confirmed occurrence of *Phlebotomus* *(Larroussius)* *orientalis* (Parrot, 1936) in Israel and place the Israeli material in a comparative phylogeographic context.

**Methods:**

Entomological surveys by CO_2_ trapping were conducted in the Negev Desert, southern Israel, between 2020 and 2024. Morphological sand fly identification was confirmed by sequencing fragments of the mitochondrial *COI* and *Cytb*/*NADH1* genes. For a newly reported species, we inferred intraspecific phylogenetic relationships and divergence times between major clades. A subset of females was additionally screened for *Leishmania* DNA and vertebrate blood-meal sources by real-time polymerase chain reaction (PCR) coupled with high-resolution melt analysis.

**Results:**

Targeted surveys and routine surveillance in the Negev region between 2020 and 2024 yielded 269 *Phlebotomus* *orientalis* (96 males, 173 females) among other species of local sand fly fauna from multiple wadi systems in the central Negev. These detections constitute the first confirmed records of *Ph.* *orientalis* in Israel. Species identification was confirmed through both morphological examination and molecular analyses of partial *COI* and *Cytb*/*NADH1* genes. Phylogenetic analysis indicated that the Israeli *Ph.* *orientalis* specimens constitute a distinct lineage that diverged from East African conspecifics during the Early to Middle Pleistocene. Blood-meal analysis of engorged *Ph.* *orientalis* females identified the European hare as a vertebrate host, and none of the tested *Ph.* *orientalis* specimens were positive for *Leishmania* DNA.

**Conclusions:**

*Phlebotomus* *orientalis* is a confirmed vector of *L.* *donovani*, the main agent of visceral leishmaniasis in East Africa. Its detection as a distinct and apparently long-established lineage in the Negev, in a region where parasites of the *L.* *donovani* complex are already involved in cutaneous leishmaniasis transmission, highlights the need to clarify the distribution, ecology, and host preferences of *Ph.* *orientalis* in Israel. Further studies are required to characterize its spatial and seasonal occurrence, evaluate its vector competence for *L.* *donovani* and *L.* *infantum*, and assess its potential contribution to current and future leishmaniasis transmission risks.

**Supplementary Information:**

The online version contains supplementary material available at 10.1186/s13071-026-07358-5.

## Background

Phlebotomine sand flies (Diptera: Psychodidae) are the primary vectors of sand fly-borne diseases (SFBDs), transmitting medically important phleboviruses (family *Phenuiviridae*, e.g., Toscana virus and sandfly fever Sicilian virus) and protozoan parasites of the genus *Leishmania*, the etiological agents of leishmaniasis [1, 2]. This neglected tropical disease remains widely endemic and climate-sensitive, affecting human and animal health in zoonotic settings [3, 4]. Recent studies have revealed an increasing climate suitability in certain regions of the Mediterranean Basin, highlighting the need for accurate, evidence-based documentation of vector assemblages and range boundaries to facilitate risk assessment [4–6].

Clinical manifestations are diverse, ranging from cutaneous leishmaniasis (CL) to visceral leishmaniasis (VL). In Israel, four *Leishmania* species have been identified: *Leishmania* *tropica*, *Leishmania* *major* (agents of CL), and *Leishmania* *infantum* (agent of VL) [7, 8]. Recently, *Leishmania* *donovani* has also been implicated in autochthonous CL transmission cycles in Israel, suggesting that the diversity of circulating parasite–vector systems may be broader than previously recognized [9].

As of now, 14 species of sand flies have been documented in Israel. Three vectors of CL have been confirmed: *Phlebotomus* *(Phlebotomus)* *papatasi* (Scopoli, 1786), the vector of *L.* *major*, and *Phlebotomus* *(Paraphlebotomus)* *sergenti* (Parrot, 1917)  as well as *Phlebotomus* *(Adlerius)* *arabicus* (Theodor, 1953), vectors of *L.* *tropica*. *Leishmania* *infantum* is believed to be transmitted by *Larroussius* sand flies, including *Phlebotomus* *(Larroussius)* *perfiliewi* *galilaeus* Theodor, 1958; *Phlebotomus* *(Larroussius)* *tobbi* (Adler, Theodor, and Lourie, 1930), and *Phlebotomus* *(Larroussius)* *syriacus* (Adler and Theodor, 1931); while *Phlebotomus* *(Paraphlebotomus)* *alexandri* (Sinton, 1928) has been suggested as the probable vector for *L.* *donovani* [7, 10–12]. Clarifying the composition and distribution of the *Larroussius* assemblage is therefore central to understanding transmission potential and prioritizing surveillance in Israel and the Eastern Mediterranean. Accurate resolution of country-level records is important because morphological overlap within *Larroussius* can confound routine identifications.

Sand fly surveillance has a long history in Israel. Since 2008, the Israeli Ministry of Health and the Ministry of Environmental Protection have coordinated systematic, nationwide, periodic surveys, progressively expanding coverage to localities without prior entomological information. The central southern Negev, an arid desert with heterogeneous habitats, was prioritized because its unique environmental conditions may support diverse sand fly populations.

*Phlebotomus* *(Larroussius)* *orientalis* (Parrot, 1936) is epidemiologically notable as a proven vector of *L.* *donovani* in East Africa, where it sustains multiple VL foci [13–16]. The taxon was originally described as *Phlebotomus* *langeroni* *var.* *orientalis* (Parrot, 1936) and was later elevated to species rank by Parrot and Clastrier (1946). It was first described from the Dire Dawa area in Ethiopia and is now reported from several countries in East and Northeast Africa and the Arabian Peninsula, including Sudan, South Sudan, Ethiopia, Eritrea, Kenya, Chad, Saudi Arabia, and Yemen [13–15]. Across these foci, *Ph.* *orientalis* is typically associated with warm, seasonally dry, semi-arid to arid landscapes characterized by *Acacia*–*Balanites* woodland and cracking clay vertisols (“black cotton soils”), which are thought to provide key breeding sites. Populations often peak in the warm dry season, with adults active in both extra-domestic thickets and peridomestic habitats [13, 14, 17, 18].

Here, we provide the first confirmed record of *Ph.* *orientalis* in Israel, based on adults collected in the central Negev between 2020 and 2024 during initial exploratory surveys and subsequent national surveillance campaigns. We describe sand fly assemblages at *Ph.* *orientalis*-positive sites and place *Ph.* *orientalis* from Israel in a comparative phylogeographic framework, aiming to determine whether the species is a recent invader of the region or a previously overlooked component of the local sand fly fauna. This baseline will support targeted follow-up on seasonality, habitat use, and host-seeking behavior under local conditions.

## Methods

### Study area

Sampling covered a south-eastern transect of southern Negev, Israel, from the Makhtesh Ramon erosion cirque (centered approximately 30.6° N, 34.8° E; 600–900 m above sea level [ASL]) across the Wadi Nekarot basin to the Wadi Paran River system along the Arava sector (centered approximately 30.3° N, 35.1° E; 150–350 m ASL).

The transect spans from the Negev highlands erosion cirques down to the Arava Valley graben, crossing contrasting lithologies and fluvial systems. Across this sector, landscapes consist of hyperarid desert steppe dissected by broad ephemeral wadis, with sandstone and limestone outcrops mantled by loessial deposits. Mean annual temperatures are warm (on the order of 20–22 °C), with hot summers frequently exceeding 35 °C and mild winters. Mean annual rainfall is very low (only a few tens of millimeters), falls mainly during winter storms, and generates short-lived flash floods in the wadis. The Nekarot and Paran sectors are characterized by ephemeral wadi systems with extensive alluvial fans, gravelly and sandy banks, and scattered *Acacia* and *Tamarix* groves; at Wadi Paran, the main channel is broad and braided. Sampling locations were selected at ecotones, including wadi margins, alluvial fans, and rocky footslopes, focusing on sheltered microhabitats such as shrub bases, boulder edges, and soil cracks typical of hyperarid channels [19].

### Trapping methods and sand fly surveillance

Sand flies were collected using Centers for Disease Control and Prevention (CDC) suction traps in two configurations, deployed overnight: (i) modified miniature traps without light, baited with dry ice as a CO_2_ source, in an updraft orientation with inlets positioned ~10 cm above ground and powered by two AA batteries [20, 21]; and (ii) CDC light traps (6 V, 150 mA; John W. Hock, Model 512, Gainesville, FL, USA) in downdraft orientation, suspended ≥ 1 m above ground from branches or fixed supports [22].

Trapping locations were selected within the ecotones described above, and coordinates and elevation were recorded at deployment using the national surveillance GIS system based on ArcGIS Pro version 3.3.2 (ESRI Inc., Redlands, CA, USA).

The sampling effort was not standardized across different years or locations. The operations conducted along the Negev–Arava transect from 2020 to 2022 were focused on exploratory surveys, with traps set opportunistically to record species occurrence. In contrast, trapping in 2023–2024 was integrated into the national surveillance program at targeted Negev and Arava localities. In alignment with the initial emphasis of this work and the presence-oriented design, we summarized the data as absolute counts per species and sites rather than as standardized abundance indices (e.g., per trap night), with a significant number of females immediately assigned to *Leishmania* pools. A year-by-site breakdown of captures is provided in Supplementary Table S2.

To minimize specimen damage, captured flies were transported to the laboratory alive under cool conditions, briefly chilled, and processed immediately. Given the first-record focus of this work, trap-night totals are not emphasized, and collections are treated as presence-oriented expeditions rather than systematic surveys.

### Specimen processing and morphological species identification

Specimens were soaked in dilute detergent (soapy water) for approximately 12 h to clear soft tissues. Heads, wings, and terminal abdominal segments of both sexes were dissected and mounted on microscope slides in Hoyer’s medium for identification using published keys [23–28]. The remaining thoraco-abdominal tissues from each individual were retained in microtubes for downstream molecular characterization.

Mounted preparations were examined using a Leica DM500 compound microscope. Diagnostic structures were imaged with an Olympus BX53 microscope equipped with a DP72 digital camera. Representative images of the taxonomic characters used for identification are provided in Fig. 3.

### DNA isolation and molecular analyses

Molecular processing of *Phlebotomus* specimens followed the Medical Entomology Laboratory (Ministry of Health, Jerusalem, Israel) protocol described by Studentsky et al. [12]. Briefly, DNA was extracted from the thoraco-abdominal remainder of each adult (heads, wings, and terminal segments mounted for morphology) by tissue disruption in lysis buffer using a TissueLyser II (QIAGEN), and nucleic acids were purified on a QIAsymphony SP with the QIASymphony DSP DNA Mini Kit (192) (QIAGEN, Cat. No./ID:937,236) and a 100 µL elution.

For routine national surveillance, unfed female sand flies were screened for *Leishmania* infection in pools of up to 20 females per tube, without prior species-level identification. Pooling was performed before DNA extraction. All specimens selected for molecular characterization and sequencing in this study were processed individually.

Downstream molecular analyses for sand fly species identification, *Leishmania* infection detection, and vertebrate blood-meal source determination were performed by real-time polymerase chain reaction (PCR) followed by high-resolution melt (HRM) analysis on a Roche LightCycler 96 (Roche, Mannheim, Germany) with AccuMelt HRM SuperMix (Quanta Bioscience, Gaithersburg, USA, cat. no. 95103–012). In line with the original Medical Entomology Laboratory protocol [12], HRM-based real-time PCR assays targeted three loci: a fragment spanning the 3′ end of *cytochrome* *b*, the adjacent *tRNA*, and the 5′ end of *NADH* *dehydrogenase* *subunit* *1* (*Cytb*/*tRNA*/*NADH1*; 368–393 base pairs [bp]) for sand fly species identification, the *internal* *transcribed* *spacer* *subunit* *I* (ITS1-219F/ITS1-219R) locus for *Leishmania* screening, and a *12S/16S* *rRNA* gene fragment (N12-16F/N12-16R) for vertebrate blood-meal source characterization (primer sets are provided in Supplementary Table S2).

For the present study, mitochondrial *cytochrome* *oxidase* *subunit* *I* (*COI*; ~700 bp) and an extended *Cytb*/*tRNA*/*NADH1* fragment were additionally amplified for sequencing-based confirmation of *Ph.* *orientalis*. To improve phylogenetic resolution within the *Larroussius* complex and to ensure compatibility with longer reference sequences available in GenBank, a new forward primer (Cytb81F) was designed in this study to amplify an extended *Cytb/tRNA/NADH1* fragment (~700 bp). Primer design was based on conserved regions identified through alignment of publicly available *Ph.* *orientalis* mitochondrial sequences. The new primer was used in combination with the previously published Cytb-R reverse primer. Primer sequences are provided in Table 1, and detailed PCR conditions are provided in Supplementary Table S1.

**Table 1:** Molecular markers and primers used in this study

Application	Target locus	Primers names	Sequence (5′–3′)	Product size
*Leishmania* detection	Internal transcribed spacer 1 locus (*ITS1*)	ITS1-219F	5′-AGCTGGATCATTTTCCGATG-3′	265 bp
		ITS1-219R	5′-ATCGCGACACGTTATGTGAG-3′	
Sand fly species identification	*Cytochrome* *b* gene (*Cytb/NADH1* = *Cytb*)	Cytb-F (short)	5′-GGAGGAGTAATYGCHYTTGTWATATC-3′	~368–393 bp
		Cytb81-F (long)	5′-AGGGTTTGCTGTTGATAATG-3′	~700 bp
		Cytb-R	5′-AAGATATTTACC**W**GCTTCKTTATGTT-3′	
	*Cytochrome* *c* *oxidase* *subunit* *I* *(COI)*	COI-F	5′-ATTCAACCAATCATAAAGATATTGGAAC-3′	~700 bp
		COI-R	5′-AAACTTCTGGATGTCCAAAAAATCAAAA-3′	
Blood-meal analysis	*12S* and *16S* genes	N12-16F	5′-ACAYACCGCCCGTCACCCTC-3′	500 bp
		N12-16R	5′-AACCAGCTATCACMAGGCTCG-3′	

Detailed PCR cycling conditions are provided in Supplementary Table S2

Degenerate bases follow International Union of Pure and Applied Chemistry (IUPAC) nomenclature

Final confirmation of *Ph.* *orientalis* identifications relied on Sanger sequencing of *COI* and the longer *Cytb*/*NADH1* fragment. PCR products were purified and submitted for sequencing at the Center for Genomic Technologies (Hebrew University of Jerusalem); bidirectional reads were assembled in BioNumerics version 8.0 (Applied Maths) and queried against GenBank using BLASTn. Sequences from Israeli *Ph.* *orientalis* generated in this study are available under accession nos. PX496653–PX496671 for *COI* and PX523241–PX523259 for *Cytb*/*NADH1*.

### Patterns of intraspecific genetic diversity

For phylogenetic and diversity analyses, the comparative dataset comprised (i) publicly available *Ph.* *orientalis* sequences retrieved from GenBank, (ii) newly generated sequences from Israel (this study), and (iii) comparative Ethiopian material from northern Ethiopia provided by Prof. Alon Warburg (the Hebrew University of Jerusalem) and available under accession nos. PX496636–PX496652 (*COI*) and PX523228–PX523240 (*Cytb*/*NADH1*) (Supplementary Table S3). DNA was processed and sequenced as described above.

All sequences (GenBank, Israel, Ethiopia) were aligned with ClustalX [29], edited in GeneDoc 2.7 [30], and translated to amino acids to confirm intact reading frames and the absence of internal stop codons. The final alignments comprised 52 *COI* and 52 *Cytb/NADH1* sequences, with lengths of 658 bp and 660 bp, respectively; missing nucleotides were coded as “N.”

Uncorrected pairwise *p*-distances were calculated in MEGA X [31]. Haplotype (*H*_d_) and nucleotide diversity (*π*) were inferred in PopART [32]. Phylogenetic analyses were conducted separately for *COI* and for *Cytb/NADH1* because many published samples include only one of these markers. Analyses were also performed on a concatenated dataset, which unavoidably contained missing data. Many published sequences were shorter than those generated in this study.

Phylogenetic inference was performed using a maximum-likelihood tree search with node support assessed via standard bootstrap analysis (1000 bootstrap replicates), in the IQ-TREE 1.6.8 plugin [33] within the PhyloSuite 1.2.2 platform [34]. The best-fitting substitution models (TIM2 + I for *COI*; TPM2 + I for *Cytb/NADH1*) were selected on the basis of the Bayesian information criterion (BIC) in ModelFinder [35]. For the concatenated dataset, PartitionFinder 2 [36], as implemented in PhyloSuite, was used to jointly infer the best partitioning scheme and substitution model (one single partition, GTR + I). Phylogenetic trees were rooted using *Ph.* *perfiliewi*, *Ph.* *syriacus*, and *Ph.* *tobbi* (GenBank accession nos. KF483665, KF483674, and KF483675 for *COI*; KC329641, KC329644, and KC329645 for *Cytb/NADH1*), in accordance with previously published tree topologies [37–39]. To further explore *Ph.* *orientalis* haplotype relationships, statistical parsimony networks [40] were constructed separately for each marker using 52 sequences per locus, after trimming alignments to match the shortest common region within each dataset (*COI*, 658 bp; *Cytb/NADH1*, overlapping fragment shared by all sequences), so as to exclude terminal sites with missing data.

To estimate the timing of major divergence events within *Ph.* *orientalis*, a time-calibrated phylogeny was inferred from combined *COI* and *Cytb/NADH1* sequence data in BEAST version 2.7.3 [41]. Two independent Markov chain Monte Carlo (MCMC) chains were run for 25 million generations each, with sampling every 1000 generations. Substitution model averaging was conducted using bModelTest [42]. A strict molecular clock was used, appropriate for intraspecific datasets [43], with minimum and maximum substitution rates of 1.0% and 2.5% per million years (MY), respectively, for the *COI* marker, covering the range typically applied to mitochondrial protein-coding genes in insects [44–47]. A Bayesian skyline coalescent tree prior [48] was used, while all other priors were left at default settings. Substitution rates for the *Cytb/NADH1* marker were estimated during the analysis. The first 10% MCMC generations from each run were discarded as burn-in, and the remaining trees and log files were combined using LogCombiner (part of the BEAST 2 package). Convergence and effective sampling of parameters were assessed in Tracer version 1.6 (available at http://beast.bio.ed.ac.uk/tracer), with all effective sample sizes (ESS) exceeding 200, indicating adequate mixing and convergence [49]. Divergence time estimates were summarized using TreeAnnotator to generate a maximum clade credibility (MCC) tree, which was visualized in FigTree version 1.4.1 (available at http://beast.bio.ed.ac.uk/figtree).

## Results

### Sand fly collections and assemblages at *Ph. orientalis*-positive sites

**Fig. 1:**
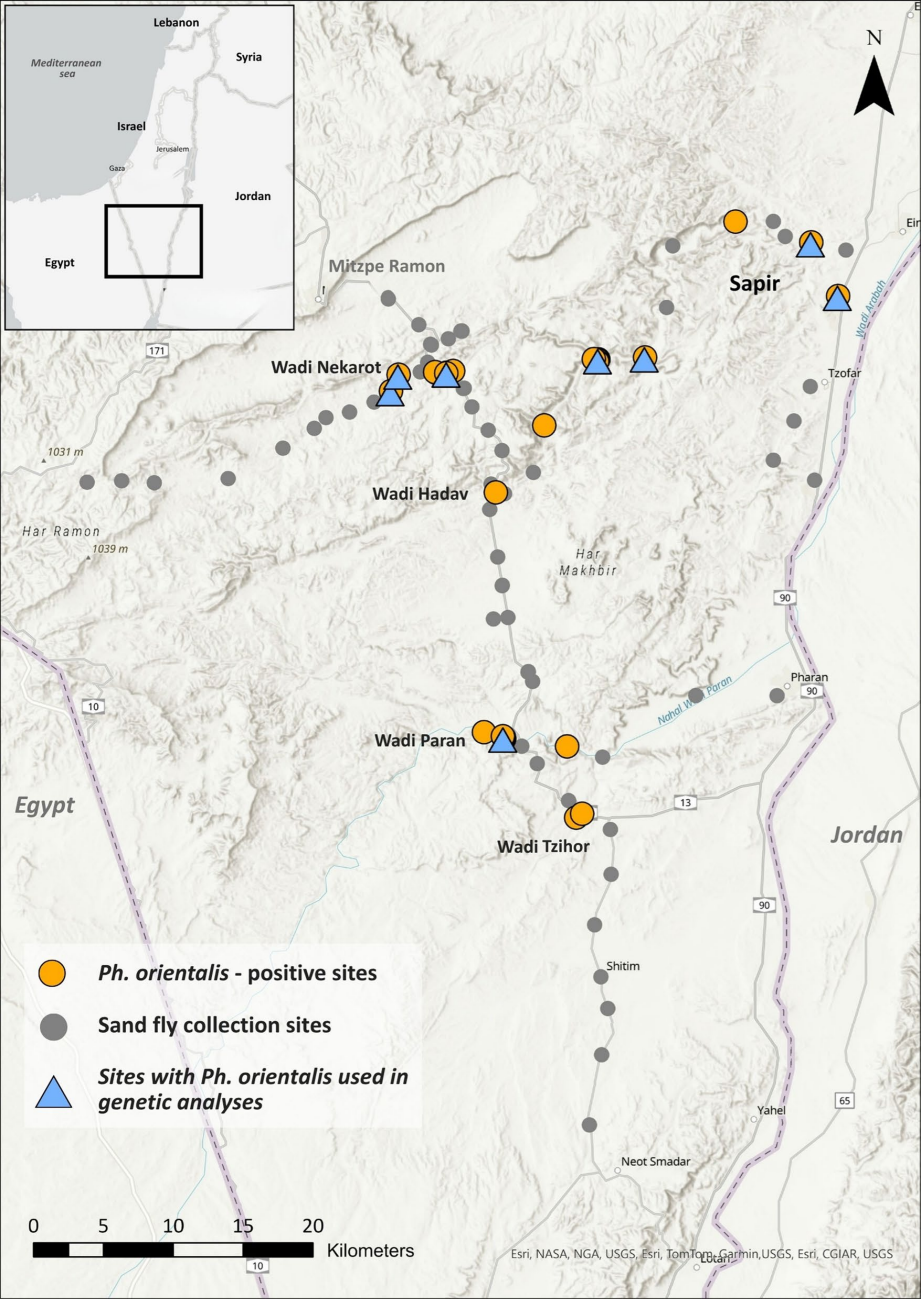
Sand fly surveillance coverage and *Phlebotomus* *orientalis* detections in the southern Negev–Arava region, Israel. Gray circles indicate all sand fly surveillance sites sampled in 2020–2024. Orange circles mark sites where *Ph.* *orientalis* was detected. Blue triangles denote *Ph.* *orientalis*-positive sites from which specimens were included in genetic analyses. Major wadis and localities (Wadi Nekarot [Nahal Nekarot], Wadi Paran [Nahal Paran], Wadi Tzihor [Nahal Tzihor], and Sapir) are labeled. Where symbols overlap, *Ph.* *orientalis* detections and genetic sampling sites are plotted above the surveillance layer. An inset map shows the position of the study area within Israel. Base map: topographic layers from Natural Earth (public domain, https://www.naturalearthdata.com/); map compiled in ArcGIS Pro 3.3.2 (Esri, Redlands, CA, USA)

*Phlebotomus* *orientalis* was detected at five sites in the central Negev (Fig. 1). Between 2020 and 2024, trapping at these localities yielded 269 *Ph.* *orientalis* adults (96 males, 173 females), with most specimens collected at Wadi Nekarot (219 individuals) and additional captures from Wadi Paran (44), Wadi Hadav (1), Sapir (3), and Wadi Tzihor (2) (Table 2 and Supplementary Table S2).

**Table 2:** Sand fly species composition at *Phlebotomus* *orientalis*-positive sites in the central Negev, Israel (2020–2024)

Species	Wadi Hadav	Wadi Nekarot	Wadi Paran	Sapir	Wadi Tzihor	Total
	N (M/F)	N (M/F)	N (M/F)	N (M/F)	N (M/F)	N (M/F)
*Ph.* *orientalis*	1 (1/0)	219 (58/161)	44 (37/7)	3 (0/3)	2 (0/2)	269 (96/173)
*Ph.* *alexandri*	143 (49/94)	2747 (1880/867)	1472 (1075/397)	0 (0/0)	2724 (1914/810)	7086 (4918/2168)
*Ph.* *kazeruni*	3 (3/0)	338 (197/141)	41 (19/22)	4 (2/2)	27 (17/10)	413 (238/175)
*Ph.* *papatasi*	0 (0/0)	25 (19/6)	92 (65/27)	33 (12/21)	493 (431/62)	643 (527/116)
*Ph.* *sergenti*	1 (0/1)	75 (43/32)	21 (12/9)	3 (0/3)	21 (18/3)	121 (73/48)
*Ph.* *syriacus*	1 (0/1)	84 (30/54)	2 (0/2)	0 (0/0)	0 (0/0)	87 (30/57)
*Ph.* *tobbi*	0 (0/0)	1 (0/1)	1 (0/1)	0 (0/0)	0 (0/0)	2 (0/2)
*Sergentomyia* spp.	85 (20/65)	45 (16/29)	179 (83/96)	0 (0/0)	231 (19/212)	540 (138/402)
Not identified (pooled)^*^	14 (0/14)	3070 (0/3070)	1592 (0/1592)	34 (0/34)	6988 (0/6988)	11,698 (0/11,698)

Values represent the absolute number of adults per species and site, expressed as *N* (M/F), where *N* is the total number of specimens, and M and F denote the numbers of males and females, respectively. “Not identified” denotes females that were not individually identified to species because they were assigned directly to pools for *Leishmania* testing within the national surveillance program

^*^ Pooled samples: each pool contained *n* = *X* specimens (or *X*–*Y* specimens)

In total, 20,859 sand flies were collected at these sites (14,839 females; 6020 males). Of these, 9161 adults (43.9%) were identified to species level, whereas 11,698 females (56.1%) were assigned directly to unidentified pools for *Leishmania* testing under the national surveillance protocol. Among identified specimens, the assemblage was dominated by *Ph.* *alexandri* (7086), with additional phlebotomine species including *Ph.* *papatasi* (643), *Ph.* *kazeruni* (413), *Ph.* *sergenti* (121), *Ph.* *syriacus* (87), and *Ph.* *tobbi* (2); *Sergentomyia* spp. were also common (540) (Table 2). Overall, *Ph.* *orientalis* represented a small fraction of the identified sand flies (2.9%) and occurred consistently within a *Larroussius* assemblage along the Negev–Arava transect.

**Fig. 2:**
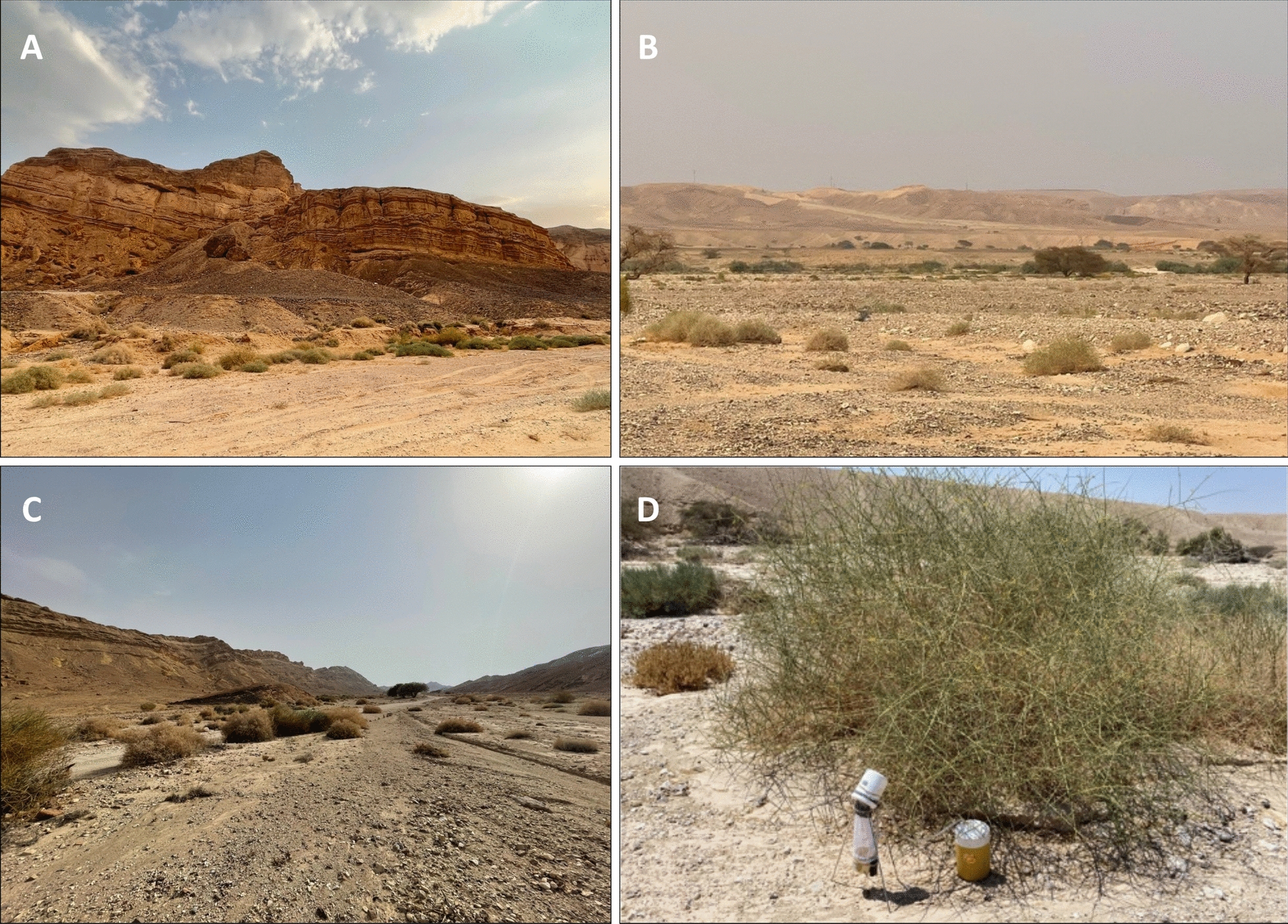
Representative *Ph.* *orientalis* habitats in the central Negev. **A** Steep bedrock wall and rubble slopes bordering the Wadi Paran floor with sparse shrub cover. **B** Broad gravelly alluvial plain with scattered shrubs and *Acacia* trees along the Arava sector. **C** Wadi channel with alluvial benches and low shrub vegetation providing shaded, sheltered microhabitats. **D** Inverted CDC light trap positioned beneath a shrub along the wadi margin at a *Ph.* *orientalis*-positive site

Positive localities for *Ph.* *orientalis* were distributed along the Wadi Nekarot River canyon system and the Wadi Paran sector of the Arava (Fig. 1). These sites shared a similar microhabitat signature: wadi margins with shaded rock faces, bedrock or rubble walls, and alluvial benches with sparse shrubs, providing sheltered, relatively humid niches within an otherwise hyperarid matrix (Fig. 2). At these sites, *Ph.* *orientalis* typically co-occurred with abundant *Ph.* *alexandri* but constituted a low-to-moderate fraction of nightly catches.

### Blood-meal sources and *Leishmania* screening in *Ph. orientalis*

Sand flies were screened for vertebrate blood-meal sources and *Leishmania* DNA. Focusing on *Ph.* *orientalis*, three engorged females were collected (one each in 2021, 2022, and 2024). Blood-meal analysis provided host identifications for two of these specimens, both corresponding to European hare (*Lepus* *europaeus*); the third engorged female did not yield a reliable host assignment. All three *Ph.* *orientalis* females were negative for *Leishmania* DNA.

### Integrative confirmation of *Ph. orientalis*

**Fig. 3:**
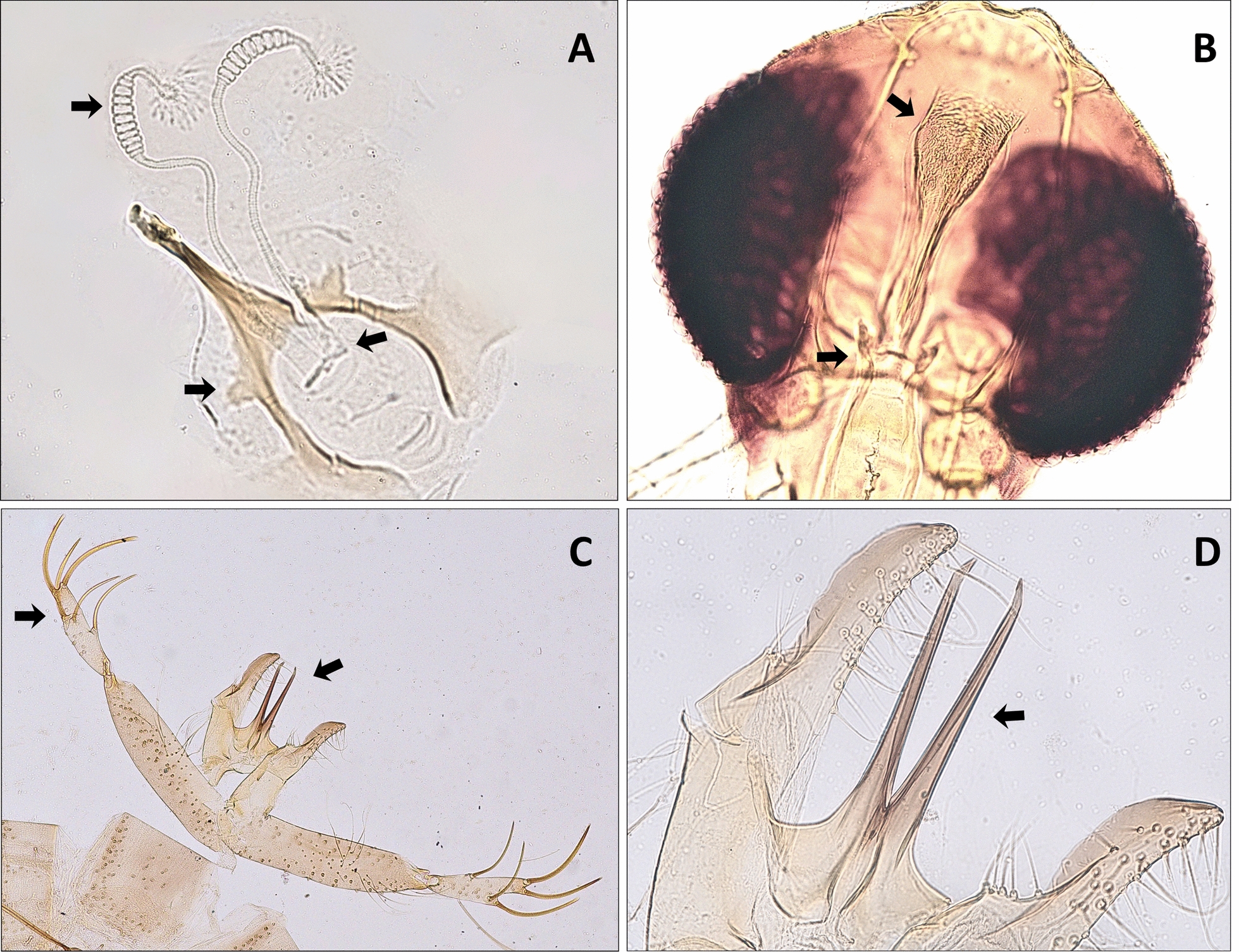
Diagnostic morphology of *Phlebotomus* *orientalis* used for species confirmation. **A** Female spermathecae: annulated individual ducts (arrow) joining a sclerotized common duct and well-defined spermathecal head (arrows). **B** cibarial and pharyngeal armature with robust median teeth and lateral denticles forming a characteristic armature pattern (arrows). **C** Male genitalia (lateral view): coxite with terminal setae (arrow) and style bearing five strong spines (arrow). **D** Aedeagus: bilobed aedeagal shaft with elongate terminal filaments (arrow). Specimens were cleared and mounted in Hoyer’s medium; images were captured on an Olympus BX53 with a DP72 camera

All *Ph.* *orientalis* records were confirmed by concordant morphological and molecular evidence. Morphological characters were consistent with published descriptions of *Ph.* *orientalis*, with males exhibiting a bilobed aedeagus bearing long terminal filaments and females multi-annulated spermathecae with a sclerotized common duct (Fig. 3). In females, we also observed the characteristic band of short, non-flattened spines in the genital atrium described by relevant taxonomical keys, as was cited in the Methods (Fig. 3).

**Fig. 4:**
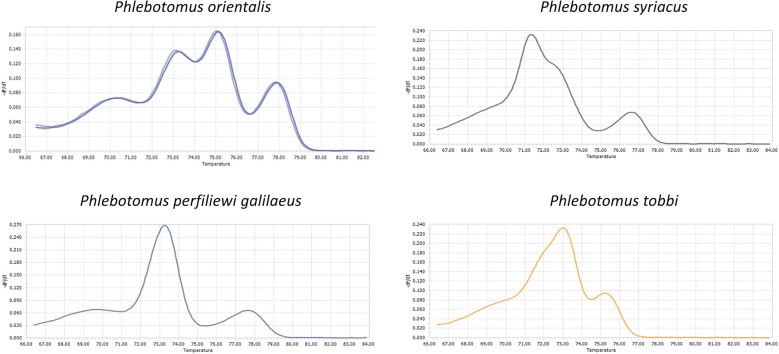
High-resolution melt (HRM) profiles of the *Cytb*/*NADH1* amplicon for *Phlebotomus* *orientalis* and other *Larroussius* species. Derivative melt curves (−dF/dT versus temperature) for *Ph.* *orientalis* (dark-green curve) are shown overlaid with representative profiles of other *Larroussius* species (colored traces). The distinct peak pattern of *Ph.* *orientalis* allows its separation from sympatric *Larroussius* taxa and was used as an initial screen prior to sequence-based confirmation

High-resolution melt (HRM) analysis of the *Cytb/NADH1* amplicon yielded a reproducible multi-peak profile for *Ph.* *orientalis* that clearly differed from the simpler melt signatures of other *Larroussius* species included in this study (e.g., *Ph.* *perfiliewi* *galilaeus*, *Ph.* *syriacus*, *Ph.* *tobbi*; Fig. 4), and all specimens assigned to *Ph.* *orientalis* on morphology shared this HRM profile.

Newly generated *COI* sequences showed 98.5–99.0% identity to reference *Ph.* *orientalis* sequences in GenBank (e.g., ON714148, KC204967), and *Cytb/NADH1* sequences showed 97.8–98.0% identity (e.g., AF161202, AF161203).

### Molecular analyses of *Phlebotomus orientalis*

**Fig. 5:**
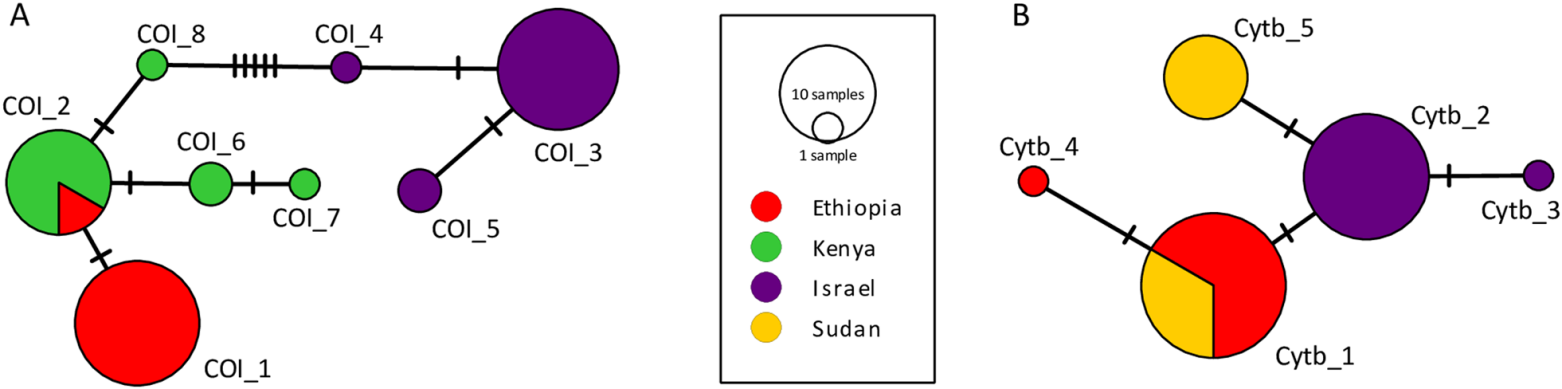
Statistical parsimony networks of *Ph.* *orientalis* based on *COI* (**A**), and *Cytb/NADH1* (**B**) sequences

Overall, eight *COI* haplotypes of *Ph.* *orientalis* were identified from all analyzed countries, comprising ten variable sites (eight parsimony-informative) with a haplotype diversity (Hd) of 0.7557, a nucleotide diversity (*π*) of 0.007, and a mean within-group distance of 0.8% (standard error [SE] = 0.2%). Two haplogroups were identified: haplogroup I comprising sequences originating from Ethiopia (*COI*_1, *COI*_2) and Kenya (*COI*_2, *COI*_6–*COI*_8), and haplogroup II comprising only sequences originating from Israel (*COI*_3–*COI*_5) (Fig. 5A). A mean pairwise distance of 1.4% (SE = 0.4%) between haplogroups and mean within-group distances of 0.2% (SE = 0.1%) and 0.07% (SE = 0.04%) were calculated in haplogroups I and II, respectively.

For *Cytb/NADH1*, five haplotypes of *Ph.* *orientalis* were identified, comprising four variable sites (one parsimony-informative) with a haplotype diversity (Hd) of 0.6554, a nucleotide diversity (*π*) of 0.01, and a mean within-group distance of 1.2% (SE = 0.3%). Three major haplotypes were observed, namely *Cytb*_1, comprising sequences originating from Ethiopia and Sudan, *Cytb*_2, including only sequences from Israel, and *Cytb*_3, comprising sequences from Sudan only (Fig. 5B).

Phylogenetic analyses based on full sequence alignments corroborate the patterns observed in the haplotype networks. In all phylogenetic trees (*COI*, *Cytb/NADH1*, and the concatenated dataset), the samples from Israel consistently formed a distinct and divergent clade (Supplementary Figs. S1–S3). This distinctiveness was relatively well supported across all trees, despite incomplete marker coverage and variable sequence lengths in the datasets. By contrast, the phylogenetic relationships between the Israeli clade and the East African samples, as well as the branching within the East African group, received only weak or negligible support. Rooting of *Ph.* *orientalis* also appears unreliable, likely reflecting the “random outgroup effect,” when the outgroup is much more divergent than the ingroup, long branches bias the inference, and can misplace the root [50–52]. This tendency is amplified by the heterogeneous, partially complete datasets.

**Fig. 6:**
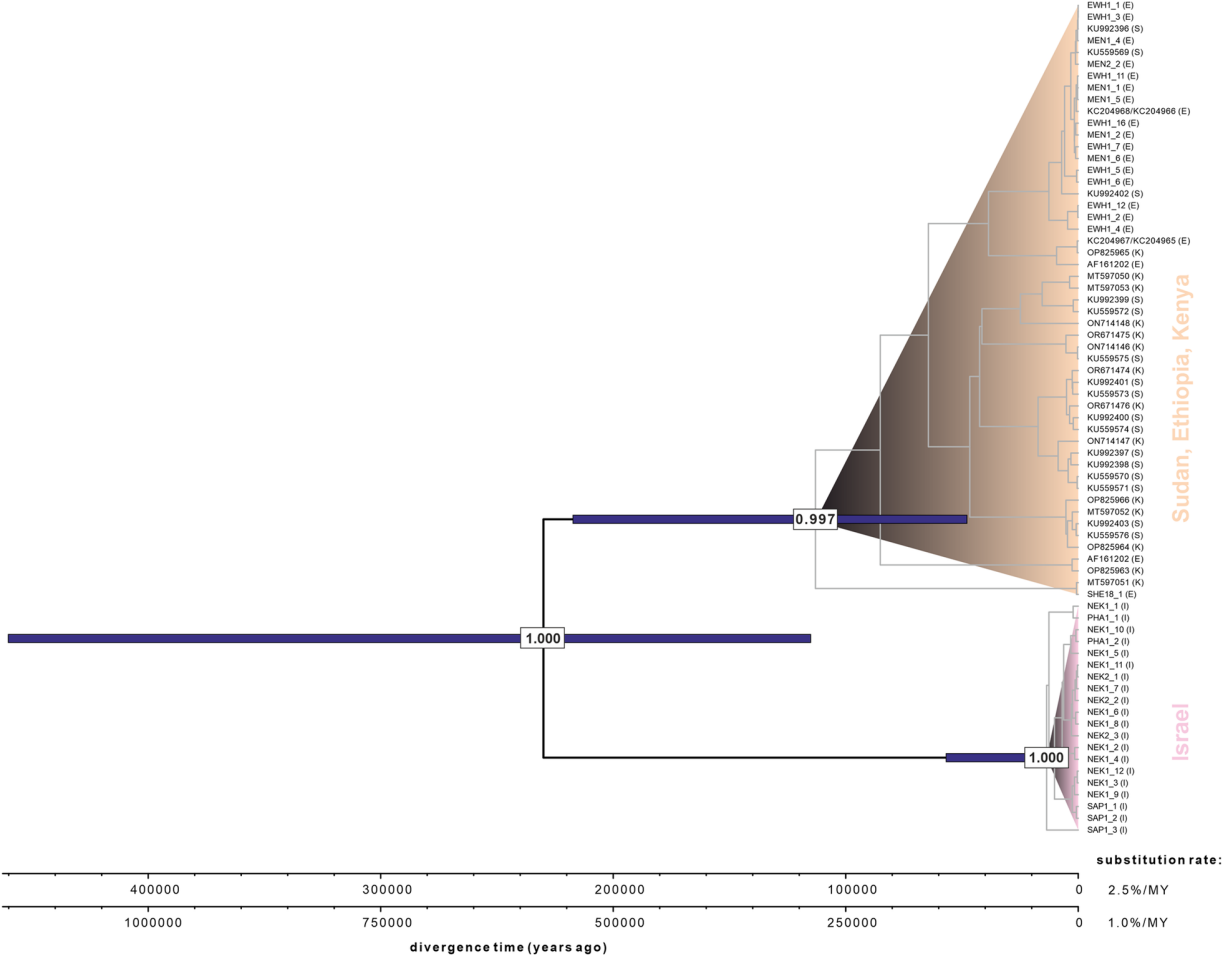
Time-calibrated tree for *Phlebotomus* *orientalis*. Blue bars indicate 95% highest posterior density (HPD) intervals for divergence time estimates at well-supported nodes (as indicated by posterior probabilities). Branching patterns within the two major lineages received little to no statistical support. Letters in parentheses refer to country of origin: Ethiopia (E); Kenya (K); Sudan (S); Israel (I)

Bayesian analysis of intraspecific divergence in *Ph.* *orientalis* using BEAST2 revealed two deeply divergent clades: an Israeli lineage and an East African lineage (Fig. 6). The reciprocal monophyly of these lineages was strongly supported. In contrast, branching patterns within each lineage received little to no support, consistent with previous phylogenetic results. Depending on the substitution rate assumed, the divergence between the two lineages occurred 230 thousand years ago (KYA; 95% highest posterior density [HPD]: 117–458 KYA) to 575 KYA (95% HPD: 293–1145 KYA). The estimated time to the most recent common ancestor (MRCA) for the Israeli lineage was dated to 13 KYA (95% HPD: 7–56 KYA) to 33 KYA (95% HPD: 18–140 KYA), while the MRCA for the East African lineage was estimated between 115 KYA (95% HPD: 48–217 KYA) and 288 KYA (95% HPD: 120–542 KYA).

## Discussion

The first confirmed country record of a vector species in a previously uncharted region can reshape local transmission assumptions and control priorities. Here we document the first confirmed occurrence of *Ph.* *orientalis* in the Negev region of Israel, representing one of the northernmost records of this species to date. This detection in a hyperarid desert setting, together with ongoing circulation of *Leishmania* *spp*. in the wider region, underscores the need to refine sand fly surveillance beyond known endemic foci and to expand systematic surveys in putatively nonendemic areas of Israel and the Mediterranean basin [7, 11].

Across endemic areas in East Africa and the Arabian Peninsula, *Ph.* *orientalis* is frequently associated with *Acacia* *seyal* and *Balanites* *aegyptiaca* dry woodland on deeply cracking “black cotton” clay (vertisol) soils, often at the margins of agricultural fields [13, 15, 17, 50]. In Ethiopia and Sudan, the species has been reported mainly from lowland and mid-elevation sites, roughly 600–1900 m ASL, where it shows marked seasonal dynamics with increased activity during the dry, warm season. These ecological correlates point to a species that can persist in xeric, semi-arid ecotonal environments with fissured clay substrates and scattered woody vegetation [13, 18].

The Negev Desert in southern Israel provides a relevant macro- and microenvironmental template and provides a contrasting but partially analogous setting. The central and southern Negev are hyperarid, with mean annual temperatures around 18–20 °C and very low precipitation (typically ~50–120 mm/year, decreasing south-eastward). Vegetation in this sector is sparse desert steppe, dominated on slopes and interfluves by dwarf shrubs such as *Artemisia* *herba-alba*, *Zygophyllum* *dumosum*, and *Reaumuria* *negevensis*, with *Anabasis* *siriaca* and *Hammada* *scoparia* in valley bottoms, and linear *Acacia* and *Tamarix* stands confined to ephemeral channels [19]. Our *Ph.* *orientalis* records derive from remote desert wadis and footslopes between −30 and 900 m ASL along the Negev highlands–Arava gradient, far from permanent settlements. These sites include cracking fine-textured substrates on wadi floors, seasonally dry channels, shrub-dominated slopes, and irrigated plots where present. Although these microhabitats are not classical black-cotton vertisols, they share key structural features with East African foci, fissured clay-rich soils, seasonal moisture pulses, and proximity to woody vegetation, suggesting that *Ph.* *orientalis* can exploit a range of xeric, clay-rich ecotones rather than a single soil category, and indicates that targeted surveys in comparable microhabitats elsewhere in Israel may reveal additional populations.

Studies on *Ph.* *orientalis* distribution in East Africa have highlighted strong associations with environmental variables such as temperature extremes, soil type, and vegetation structure. Incorporating analogous predictors for Israel into spatial models, combined with targeted field validation in clay-rich wadis and adjacent slopes, will help to refine maps of the current distribution of *Ph.* *orientalis* and to anticipate potential range shifts under ongoing environmental change [13–15, 18].

Across the Eastern Mediterranean, multiple *Leishmania* transmission systems coexist [7, 9]. The presence of *Ph.* *orientalis* in Israel therefore has public health relevance, particularly in relation to VL. Visceral leishmaniasis in Israel is attributed to *L.* *infantum*, whereas *L.* *donovani* has thus far been documented only in cutaneous forms in the Negev. Within this epidemiological context, the detection of *Ph.* *orientalis*, a proven vector of viscerotropic *L.* *donovani* in East Africa, raises questions about its local vector competence and ecology rather than implying visceral *L.* *donovani* transmission foci. Current data from the central Negev indicate that *L.* *donovani* circulation remains confined to cutaneous manifestations associated with *Ph.* *alexandri* and the European hare as a potential reservoir, and no visualization of disease has been documented to date [7, 9, 51]. The xeric rocky wadi habitats where we detected *Ph.* *orientalis* overlap those reported for *Ph.* *alexandri* in Negev *L.* *donovani* foci, which justifies targeted, hypothesis-driven surveillance rather than immediate vector incrimination. The detection of *Ph.* *orientalis* in Israel, therefore, warrants focused risk assessment and enhanced surveillance design.

The newly observed presence of *Ph.* *orientalis* in the Negev desert and Israel in general may be attributed to two nonexclusive scenarios. Firstly, a recent introduction event, facilitated by natural climate-associated spread or anthropogenic dispersal by migration of people, animals, or commodities across different areas. Secondly, *Ph.* *orientalis* may have been present in Israel for an extended period but remained unreported, possibly owing to limited historical sampling rather than true absence. These alternative scenarios are examined using mitochondrial divergence patterns. We also consider the ecological setting of the Israeli capture sites relative to known *Ph.* *orientalis* ecotones and discuss hypotheses for establishment and persistence consistent with current evidence.

Beyond documenting a new country record, we placed the Israeli material within a comparative phylogeographic framework alongside East African reference sequences to evaluate whether the Negev population represents a recent arrival or an overlooked lineage. Phylogenetic inference in our study is based on datasets with heterogeneous sampling and limited reference sequences, but despite these limitations, our results indicate a long-standing, separate evolutionary history of *Ph.* *orientalis* in Israel. Estimated divergence of approximately 230–575 thousand years ago, calculated from mitochondrial percent divergence under 1% and 2.5% substitution rates, respectively, places separation in the Early to Middle Pleistocene. On this basis, a recent introduction from East Africa appears unlikely, although the paucity of reference sequences from adjacent regions, including Egypt and Sinai, does not allow us to exclude a nearby source population.

Sand fly species composition in the central Negev region is high, with six *Phlebotomus* species collected from ten trapping sites, with *Ph.* *alexandri*, *Ph.* *orientalis*, and *Phlebotomus* *(Paraphlebotomus)* *kazeruni* (Theodor and Mesghali, 1964) being most abundant, which aligns with a recently published study in the same area [9]. The co-occurrence of several *Larroussius* species in the Negev with the circulation of *L.* *donovani*-associated CL focus and sporadic *L.* *infantum* is epidemiologically important. In our surveys, three blood-fed *Ph.* *orientalis* females yielded vertebrate DNA consistent with European hare, while none of the screened *Ph.* *orientalis* specimens were positive for *Leishmania* DNA. This pattern indicates ecological overlap between *Ph.* *orientalis* and *Ph.* *alexandri* in local transmission settings, but the role of *Ph.* *orientalis* in the Negev *L.* *donovani* focus, if any, remains unresolved. Overall, the presence of several sympatric *Larroussius* species emphasizes that vector roles in the Negev cannot be inferred from presence alone and require species-resolved ecological and experimental studies.

The discovery of *Ph.* *orientalis* in the Negev adds to the broader *Larroussius* assemblage present in Israel and neighboring regions, which includes species such as *Ph.* *tobbi*, *Ph.* *syriacus*, and *Ph.* *perfiliewi* *galilaeus* that are confirmed or suspected vectors of *L.* *infantum*. This regional complex of closely related sand flies, some with overlapping ranges and habitats, highlights the complexity of VL transmission in the Eastern Mediterranean. The confirmed role of *Ph.* *orientalis* as a vector for *L.* *donovani* elsewhere therefore warrants further research into its ecological interactions, seasonal dynamics, and potential to transmit other pathogens.

Species identification based solely on morphological features has limitations in classifying new species [52]. Therefore, in our study, species identification was achieved through co-analysis of morphological and molecular data. Employing this integrative approach helped clarify and confirm the discovery of a distinct *Ph.* *orientalis* lineage in Israel compared with specimens from Ethiopia, Sudan, or Kenya. Available reference sequences for *Ph.* *orientalis* remain limited and are geographically uneven, so the Israeli sequences deposited in public repositories partly fill a gap for the Eastern Mediterranean. Additional sampling and sequencing from North Africa, the Arabian Peninsula, and neighboring regions such as Egypt and Sinai will be important to refine phylogeographic structure across the range and to improve reference libraries for routine molecular identification. Comparable patterns of regionally structured lineages have been reported for *Ph.* *simici*, which forms three distinct lineages in the Balkans, Turkey, and Israel, respectively [52], underscoring the value of integrative taxonomic frameworks for *Larroussius* sand flies.

## Conclusions

*Phlebotomus* *orientalis* is recorded here for the first time in Israel as part of the regional *Larroussius* assemblage. Using an integrative approach that combines morphology and mitochondrial markers, our data can support future sand fly surveillance in the region. Given the role of this species in East Africa and the recent detection of *L.* *donovani* in the same area in Israel, future entomological surveys are essential to resolve basic questions: seasonal dynamics and abundance in relevant microhabitats, host-seeking behavior, and, critically, the epidemiological implications of *Ph.* *orientalis* as a possible vector of *L.* *donovani* under local conditions. The answers will determine whether this record remains primarily faunistic or carries broader public health implications.

We present integrative evidence for *Ph.* *orientalis* in Israel and outline a conservative interpretation of its status within the regional *Larroussius* assemblage. By distinguishing clearly between what is demonstrated here and what requires further study, this work contributes to sand fly taxonomy in the Eastern Mediterranean and provides a practical baseline for risk-proportionate surveillance. Control and prevention strategies should focus on integrated vector management approaches, including environmental management, community education, and targeted use of insecticides.

## Supplementary Information

Additional file 1 (TIF 188 KB) Supplementary Figure S1. Phylogenetic relationship (ML tree) within *Phlebotomus* *orientalis* based on *COI* data. Only bootstrap support values > 50 are shown. Letters in parentheses refer to country of origin: E—Ethiopia; I—Israel; K—Kenya.

Additional file 2 (TIF 198 KB) Supplementary Figure S2. Phylogenetic relationship (ML tree) within *Phlebotomus* *orientalis* based on *Cytb*/*NADH1* data. Only bootstrap support values > 50 are shown. Letters in parentheses refer to country of origin: E—Ethiopia; I—Israel; S—Sudan.

Additional file 3 (TIF 214 KB) Supplementary Figure S3. Phylogenetic relationship (ML tree) within *Phlebotomus* *orientalis* based on concatenated (*COI* plus *Cytb*/*NADH1*) data. Only bootstrap support values > 50 are shown. Letters in parentheses refer to country of origin: E—Ethiopia; I—Israel; K—Kenya; S—Sudan.

Additional file 4 (DOCX 6179 KB) Supplementary Table S1. Designed primers and PCR conditions used in this study.

Additional file 5 (DOCX 34 KB) Supplementary Table S2. Yearly sand fly catches at Phlebotomus orientalis–positive sites in the central Negev, Israel (2020–2024). For each location, species-specific counts of males (M) and females (F) are presented by year of collection, along with a total count across all years. Elevation (m ASL) and approximate centered coordinates (WGS84) are given for each location. “Pooled” refers to females that were not individually identified to species because they were allocated directly to pools for Leishmania screening within the national surveillance program.

Additional file 6 (XLSX 19 KB)

## Data Availability

Data are provided within the manuscript or supplementary information files.

## Abbreviations

CL: Cutaneous leishmaniasis
*COI*: Cytochrome c oxidase subunit 1
*Cytb*: Cytochrome b
*NADH1*: Natrium dehydrogenase 1
SFBDs: Sand fly-borne diseases
SFSV: Sandfly fever Sicilian virus
TOSV: Toscana virus
VL: Visceral leishmaniasis
