# Supplementary figures and images for: First record of Phlebotomus (Larroussius) orientalis (Parrot, 1936) (Diptera: Psychodidae) in Israel: phylogeographic placement and implications for leishmaniasis surveillance
Source: Parasit Vectors. 2026 Mar 29;19:203. doi: 10.1186/s13071-026-07358-5 (PMC13154854; doi:10.1186/s13071-026-07358-5)

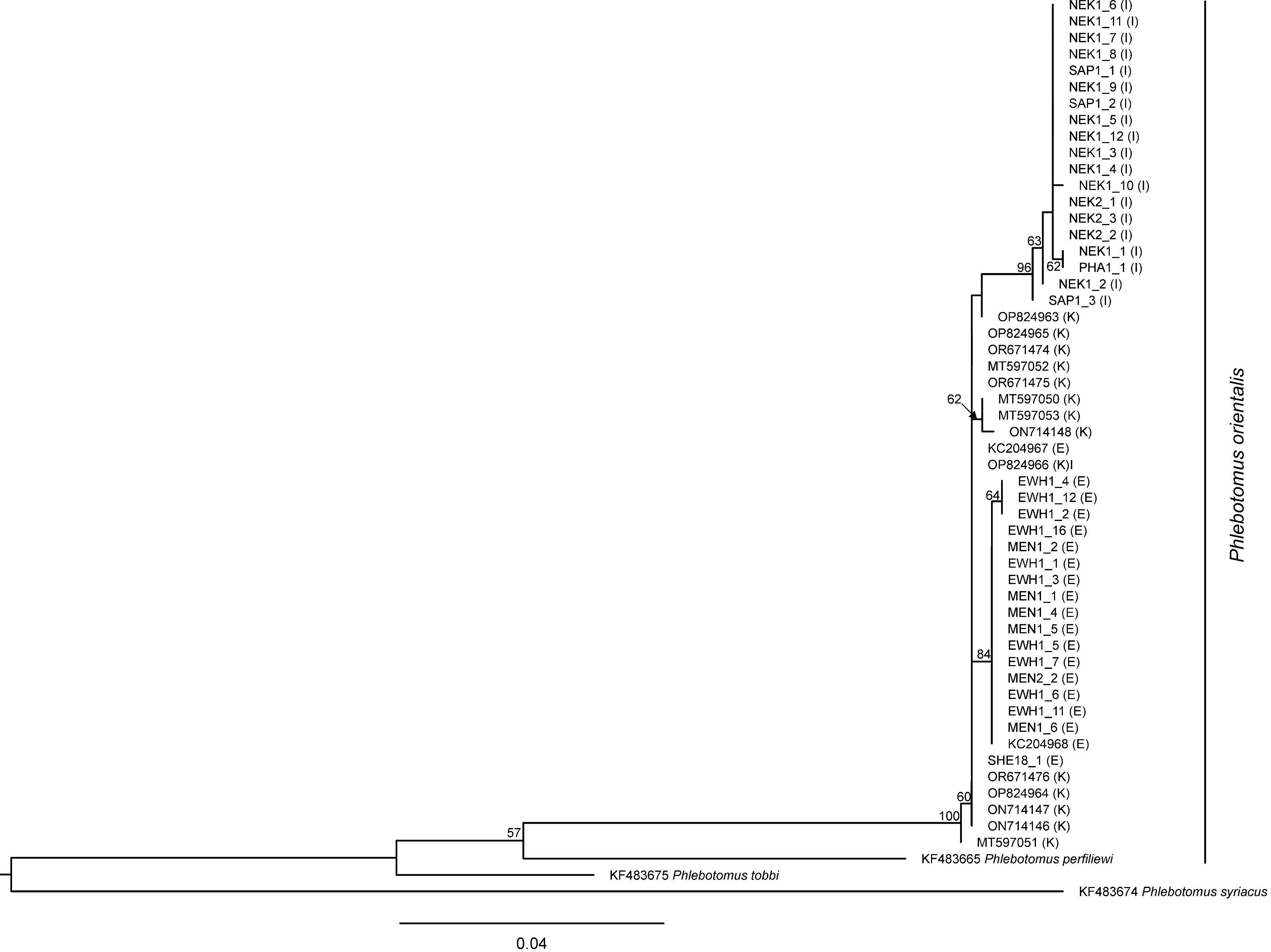

Supplement: Supplementary file 1 — Additional file 1 (TIF 188 KB) Supplementary Figure S1. Phylogenetic relationship (ML tree) within Phlebotomus orientalis based on COI data. Only bootstrap support values > 50 are shown. Letters in parentheses refer to country of origin: E—Ethiopia; I—Israel; K—Kenya. [file 13071_2026_7358_MOESM1_ESM.tif]

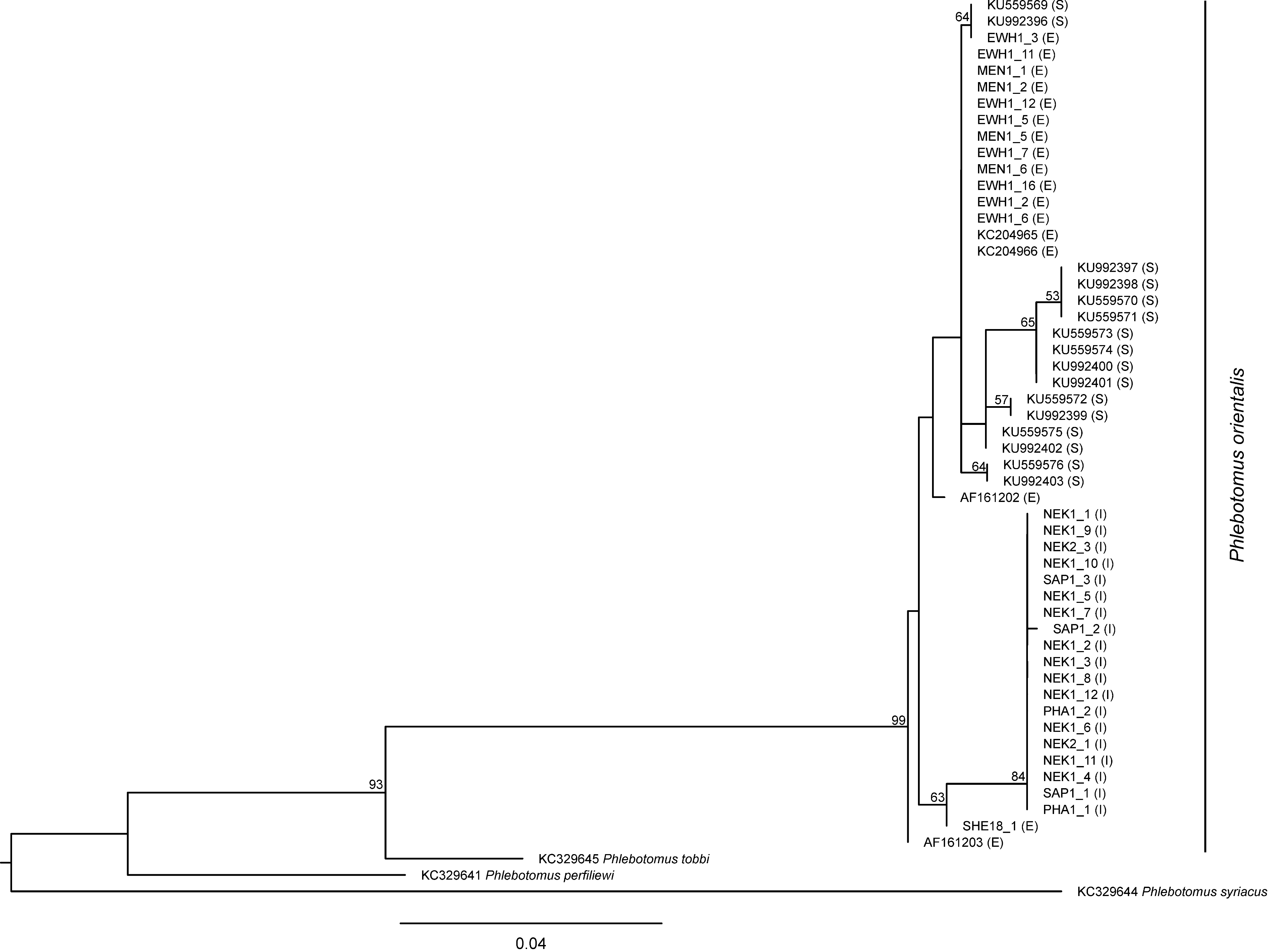

Supplement: Supplementary file 2 — Additional file 2 (TIF 198 KB) Supplementary Figure S2. Phylogenetic relationship (ML tree) within Phlebotomus orientalis based on Cytb/NADH1 data. Only bootstrap support values > 50 are shown. Letters in parentheses refer to country of origin: E—Ethiopia; I—Israel; S—Sudan. [file 13071_2026_7358_MOESM2_ESM.tif]

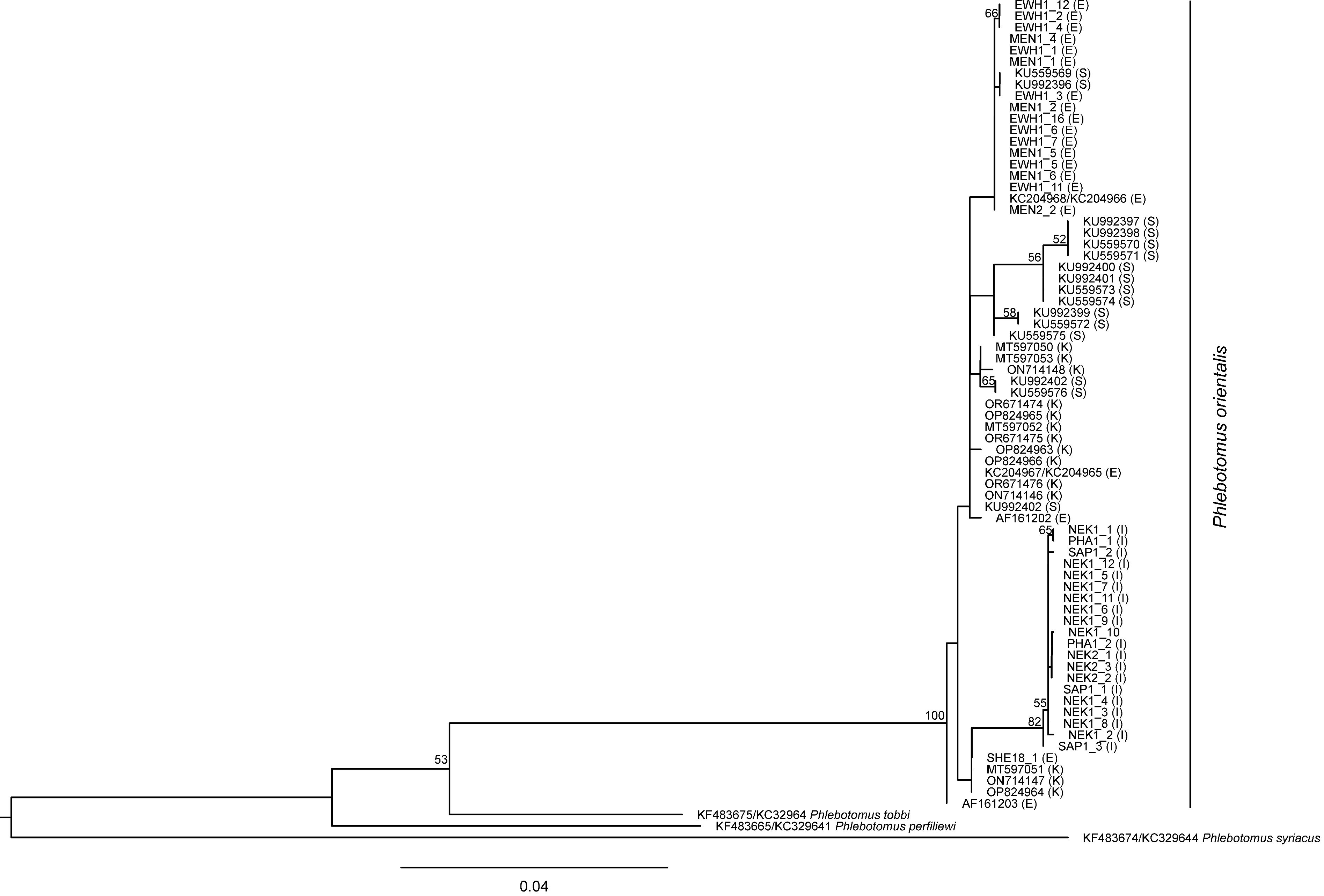

Supplement: Supplementary file 3 — Additional file 3 (TIF 214 KB) Supplementary Figure S3. Phylogenetic relationship (ML tree) within Phlebotomus orientalis based on concatenated (COI plus Cytb/NADH1) data. Only bootstrap support values > 50 are shown. Letters in parentheses refer to country of origin: E—Ethiopia; I—Israel; K—Kenya; S—Sudan. [file 13071_2026_7358_MOESM3_ESM.tif]
