# Supplementary material for: First record of Phlebotomus (Larroussius) orientalis (Parrot, 1936) (Diptera: Psychodidae) in Israel: phylogeographic placement and implications for leishmaniasis surveillance
Source: Parasit Vectors. 2026 Mar 29;19:203. doi: 10.1186/s13071-026-07358-5 (PMC13154854; doi:10.1186/s13071-026-07358-5)
Supplement: Supplementary file 4 — Additional file 4 (DOCX 6179 KB) Supplementary Table S1. Designed primers and PCR conditions used in this study. [file 13071_2026_7358_MOESM4_ESM.docx]

**Supplementary Table S1**. Designed primers and PCR conditions used in this study

| **Application** | **Target locus** | **Primers names** | **Sequences (5’ – 3’)** | **Annealing temp. (°C)** | **PCR Program** | **Product size** |
| --- | --- | --- | --- | --- | --- | --- |
| ***Leishmania* detection** | Internal transcribed spacer 1 locus (*ITS1*) | ITS1-219F | 5'-AGCTGGATCATTTTCCGATG-3' | 60°C | 95°C - 5 min; 45 cycles [95°C - 10 sec; 60°C - 45 sec]; 95°C - 60 sec; 40°C - 60 sec; 65°C - 1 sec; 97°C - 1 sec; 37°C - 30 sec. | 265 bp |
|  |  | ITS1-219R | 5'-ATCGCGACACGTTATGTGAG-3' |  |  |  |
| **Sand fly species identification** | *Cytochrome b* gene  (*Cytb/NADH1 =* Cytb) | Cytb-F (short) | 5'-GGAGGAGTAATYGCHYTTGTWATATC-3' | 38°C - 60°C | 95°C - 5 min; 45 cycles [95°C - 5 sec; 38°C/43°C - 2 sec; 60°C - 45 sec]; 95°C - 60 sec; 40°C - 60 sec; 65°C - 1 sec; 85°C - 1 sec; 37°C - 30 sec. | ~368-393 bp |
|  |  | Cytb81-F (long) | 5'-AGGGTTTGCTGTTGATAATG-3' | 43°C - 60°C |  | ~700 bp |
|  |  | Cytb-R | 5'-AAGATATTTACC**W**GCTTCKTTATGTT-3' |  |  |  |
|  | *Cytochrome c oxidase subunit I (COI)* | COI-F | 5'-ATTCAACCAATCATAAAGATATTGGAAC-3' | 38°C - 60°C | 95°C - 5 min; 45 cycles [95°C - 5 sec; 38°C - 2 sec; 60°C - 45 sec]; 95°C - 60 sec; 40°C - 60 sec; 65°C - 1 sec; 85°C - 1 sec; 37°C - 30 sec. | ~700 bp |
|  |  | COI-R | 5'-AAACTTCTGGATGTCCAAAAAATCAAAA-3' |  |  |  |
| **Blood meal source analysis** | *12S* and *16S* genes | N12-16F | 5'-ACAYACCGCCCGTCACCCTC-3' | 61°C | 95°C - 5 min; 40 cycles [95°C - 10 sec; 61°C - 45 sec]; 95°C - 60 sec; 40°C - 60 sec; 65°C - 1 sec; 85°C - 1 sec; 37°C - 30 sec. | 500 bp |
|  |  | N12-16R | 5'-AACCAGCTATCACMAGGCTCG-3' |  |  |  |

* Degenerate bases follow IUPAC nomenclature.
