# Supplementary material for: First record of Phlebotomus (Larroussius) orientalis (Parrot, 1936) (Diptera: Psychodidae) in Israel: phylogeographic placement and implications for leishmaniasis surveillance
Source: Parasit Vectors. 2026 Mar 29;19:203. doi: 10.1186/s13071-026-07358-5 (PMC13154854; doi:10.1186/s13071-026-07358-5)
Supplement: Supplementary file 5 — Additional file 5 (DOCX 34 KB) Supplementary Table S2. Yearly sand fly catches at Phlebotomus orientalis–positive sites in the central Negev, Israel (2020–2024). For each location, species-specific counts of males (M) and females (F) are presented by year of collection, along with a total count across all years. Elevation (m ASL) and approximate centered coordinates (WGS84) are given for each location. “Pooled” refers to females that were not individually identified to species because they were allocated directly to pools for Leishmania screening within the national surveillance program. [file 13071_2026_7358_MOESM5_ESM.docx]

**Supplementary Table S2. Yearly sand fly catches at *Phlebotomus orientalis*–positive sites in the central Negev, Israel (2020–2024).**

For each location, species-specific counts of males (M) and females (F) are presented by year of collection, along with a total count across all years. Elevation (m ASL) and approximate centered coordinates (WGS84) are given for each location. “Pooled” refers to females that were not individually identified to species because they were allocated directly to pools for Leishmania screening within the national surveillance program.

| **Location** | **Coordinates (Lat/Long)** | **Altitude**  **(m ASL)** | **Species** | **2020** | | **2021** | | **2022** | | **2023** | | **2024** | | **Total** | | **Grand Total** |
| --- | --- | --- | --- | --- | --- | --- | --- | --- | --- | --- | --- | --- | --- | --- | --- | --- |
|  |  |  |  | **M** | **F** | **M** | **F** | **M** | **F** | **M** | **F** | **M** | **F** | **M** | **F** |  |
| **Wadi Nekarot** | **30.566° N, 34.903° E** | **-30 - 899** | ***Ph***. ***orientalis*** |  | **4** | **17** | **92** | **9** | **41** | **6** | **13** | **26** | **11** | **58** | **161** | **219** |
|  |  |  | *Ph*. *alexandri* | 338 | 226 | 69 | 40 | 268 | 163 | 131 | 79 | 1074 | 359 | **1,880** | **867** | **2,747** |
|  |  |  | *Ph*. *kazeruni* | 11 | 8 | 2 | 17 | 89 | 67 | 93 | 46 | 2 | 3 | **197** | **141** | **338** |
|  |  |  | *Ph*. *papatasi* | 2 |  |  | 1 | 2 | 2 | 11 | 2 | 4 | 1 | **19** | **6** | **25** |
|  |  |  | *Ph. sergenti* | 13 | 6 | 6 | 5 | 16 | 9 | 4 | 4 | 4 | 8 | **43** | **32** | **75** |
|  |  |  | *Ph. syriacus* |  | 8 | 9 | 23 | 16 | 16 | 3 | 4 | 2 | 3 | **30** | **54** | **84** |
|  |  |  | *Ph. tobbi* |  |  |  |  |  |  |  | 1 |  |  | **0** | **1** | **1** |
|  |  |  | *Sergentomyia spp*. | 1 | 1 |  |  |  |  | 12 | 22 | 3 | 6 | **16** | **29** | **45** |
|  |  |  | Pooled |  | 487 | 0 | 72 |  | 215 |  | 626 |  | 1670 | **0** | **3,070** | **3,070** |
| **Wadi Paran** | **30.328° N, 34.941° E** | **118 - 320** | ***Ph. orientalis*** |  |  | **31** | **2** | **2** |  | **2** | **2** | **2** | **3** | **37** | **7** | **44** |
|  |  |  | *Ph. alexandri* | 377 | 32 | 273 | 101 | 2 | 4 | 267 | 95 | 156 | 165 | **1,075** | **397** | **1,472** |
|  |  |  | *Ph. kazeruni* | 8 |  | 8 | 7 | 2 | 3 | 1 | 11 |  | 1 | **19** | **22** | **41** |
|  |  |  | *Ph. papatasi* |  |  | 10 | 6 | 1 | 4 | 16 | 2 | 38 | 15 | **65** | **27** | **92** |
|  |  |  | *Ph. sergenti* |  |  | 4 |  |  |  | 4 | 5 | 4 | 4 | **12** | **9** | **21** |
|  |  |  | *Ph. syriacus* |  |  |  | 1 |  |  |  |  |  | 1 | **0** | **2** | **2** |
|  |  |  | *Ph. tobbi* |  |  |  |  |  |  |  |  |  | 1 | **0** | **1** | **1** |
|  |  |  | *Sergentomyia spp*. |  |  |  |  |  | 1 | 46 | 47 | 37 | 48 | **83** | **96** | **179** |
|  |  |  | Pooled |  | 428 |  | 256 | 0 | 2 |  | 578 |  | 328 | **0** | **1,592** | **1,592** |
| **Sapir** |  | **30.614° N, 34.189° E** | ***Ph. orientalis*** |  | **2** |  | **1** |  |  |  |  |  |  | **0** | **3** | **3** |
|  |  |  | *Ph. kazeruni* | 2 | 2 |  |  |  |  |  |  |  |  | **2** | **2** | **4** |
|  | **-30** |  | *Ph. papatasi* | 6 | 9 | 6 | 11 |  |  |  |  |  | 1 | **12** | **21** | **33** |
|  |  |  | *Ph. sergenti* |  | 1 |  | 2 |  |  |  |  |  |  | **0** | **3** | **3** |
|  |  |  | Pooled |  | 20 |  | 14 |  |  |  |  |  |  | **0** | **34** | **34** |
| **Wadi Tzihor** | **30.280° N, 34.000° E** | **260** | ***Ph. orientalis*** |  |  |  |  |  |  |  | **1** |  | **1** | **0** | **2** | **2** |
|  |  |  | *Ph. alexandri* | 739 | 21 |  |  |  |  | 462 | 523 | 713 | 266 | **1,914** | **810** | **2,724** |
|  |  |  | *Ph. kazeruni* |  | 2 |  |  |  |  | 8 | 8 | 9 |  | **17** | **10** | **27** |
|  |  |  | *Ph. papatasi* | 58 | 3 |  |  |  |  | 81 | 4 | 292 | 55 | **431** | **62** | **493** |
|  |  |  | *Ph. sergenti* | 15 |  |  |  |  |  | 1 |  | 2 | 3 | **18** | **3** | **21** |
|  |  |  | *Sergentomyia spp*. |  |  |  |  |  |  | 13 | 205 | 6 | 7 | **19** | **212** | **231** |
|  |  |  | Pooled |  | 1256 |  |  |  |  |  | 3613 |  | 2119 | **0** | **6,988** | **6,988** |
| **Wadi Hadav** |  | **400** | ***Ph. orientalis*** |  |  |  |  |  |  |  |  | **1** |  | **1** | **0** | **1** |
|  |  |  | *Ph. alexandri* |  |  |  |  |  |  | 13 | 40 | 36 | 54 | **49** | **94** | **143** |
|  |  |  | *Ph. kazeruni* |  |  |  |  |  |  | 1 |  | 2 |  | **3** | **0** | **3** |
|  | **30.487° N, 34.935° E** |  | *Ph. sergenti* |  |  |  |  |  |  |  | 1 |  |  | **0** | **1** | **1** |
|  |  |  | *Ph. syriacus* |  |  |  |  |  |  |  | 1 |  |  | **0** | **1** | **1** |
|  |  |  | *Sergentomyia spp*. |  |  |  |  |  |  | 18 | 50 | 2 | 15 | **20** | **65** | **85** |
|  |  |  | Pooled |  |  |  |  |  |  |  |  |  | 14 | **0** | **14** | **14** |
| **Total** | | | | **1,570** | **2,516** | **435** | **651** | **407** | **527** | **1,193** | **5,983** | **24,15** | **5,162** | **6,020** | **14,839** | **20,859** |
